# Supplementary material for: Precursor processes of human self-initiated action
Source: Neuroimage. 2018 Jan 15;165:35–47. doi: 10.1016/j.neuroimage.2017.09.057 (PMC5737384; doi:10.1016/j.neuroimage.2017.09.057)
Supplement: Supplementary data [file mmc1.pdf]

Supporting Information

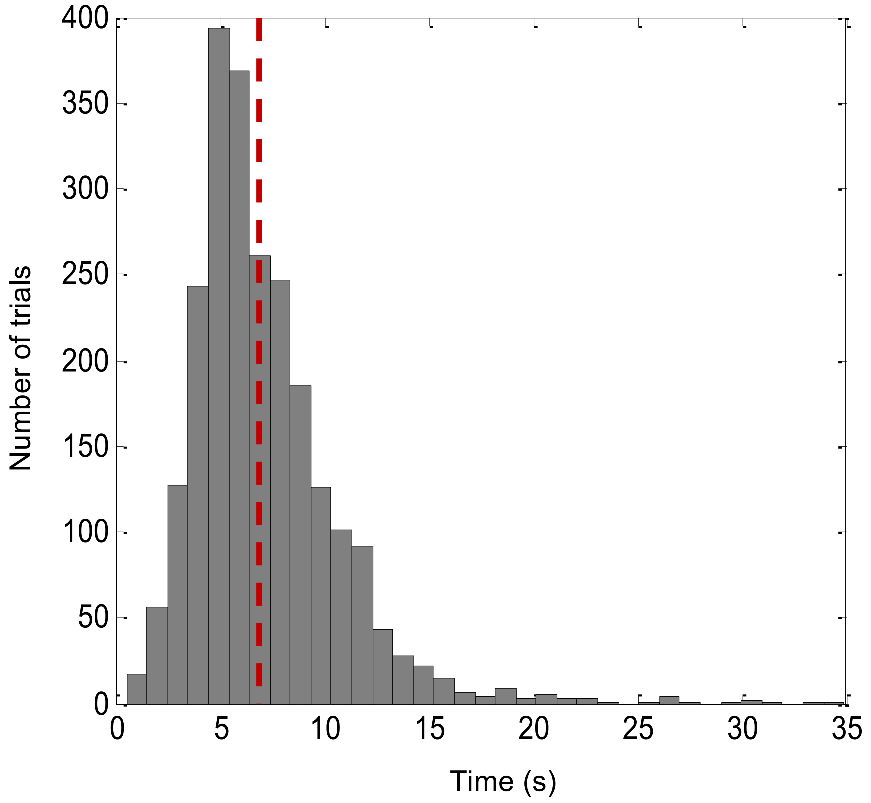

Figure S1. Histogram of waiting times before skip actions in self-initiated condition, across all trials and all participants. The dashed red line shows the average waiting time.

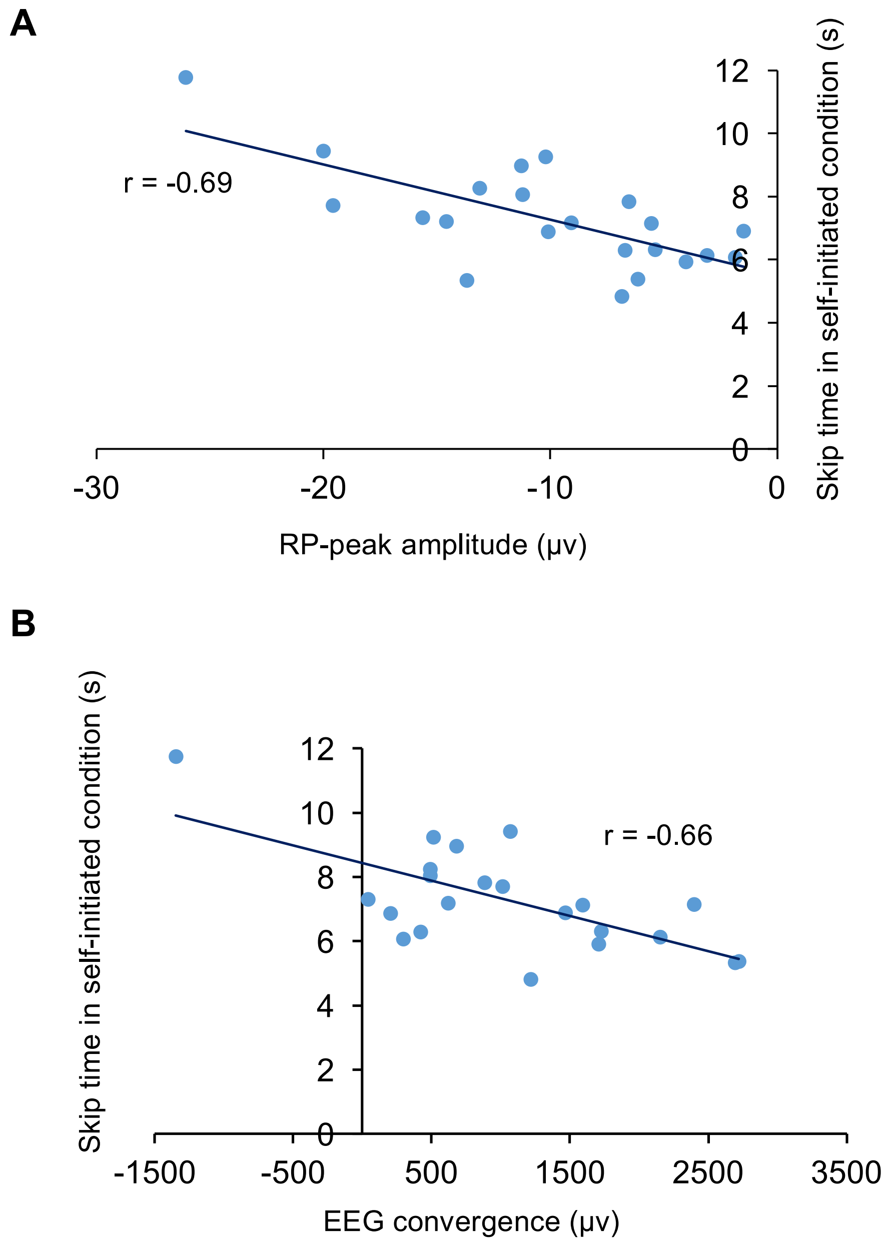

Figure S2. Correlation between participants' mean waiting time (s) before skipping in the self-initiated condition and RP-peak amplitude (A), and EEG convergence (B). EEG convergence was measured by subtracting the area under the SD curve in self-initiated from the externally-triggered condition. There was a significant negative correlation between waiting time and RP-peak amplitude (Pearson's $r = -0.69$, $p < 0.01$), and maximum EEG convergence (Pearson's $r = -0.66$, $p < 0.01$).

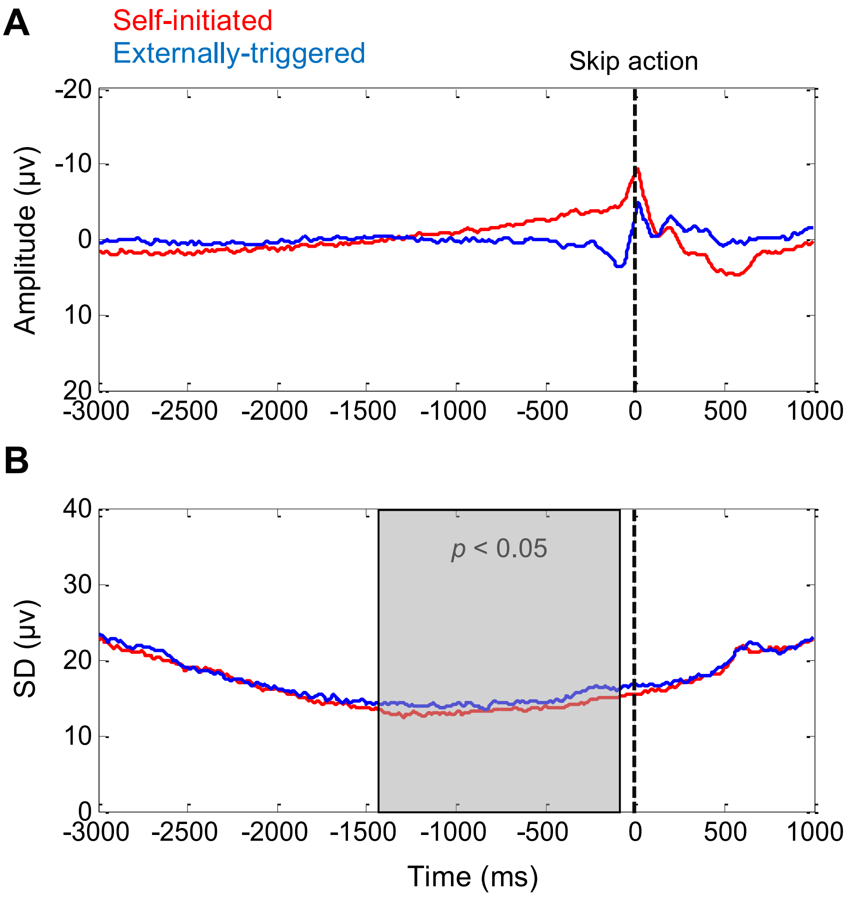

Figure S3. Demeaned EEG activity prior to skip actions. The red and blue lines represent self-initiated and externally-triggered skips, respectively. Data is time-locked to the skip action (black vertical line), and is baselined to the mean of entire epoch (i.e., demeaned), and recorded from FCz electrode. A. Grand average RP amplitude. B. Standard deviation across trials averaged across participants. Shaded area show significant clusters across central electrodes, detected by cluster-based permutation test. Whereas baselining to a limited time window forces a low SD within the baseline time window, and a progressive rise in SD with temporal distance before or after the baseline, the use of a broad baseline time window, as here, reduces this artefactual effect of baseline-correction on variability of time-locked data. Nevertheless, the difference in SD between conditions remains significant.

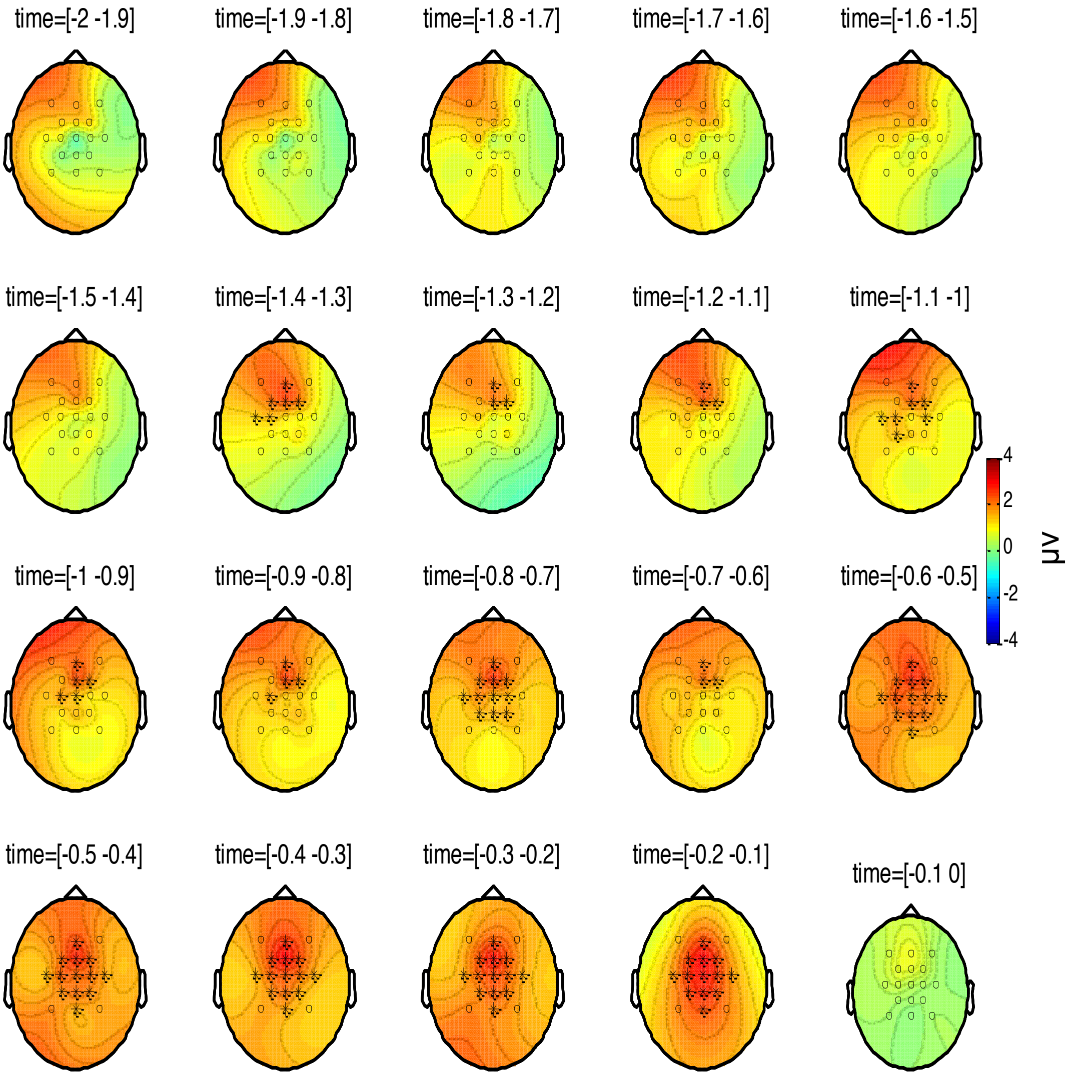

Figure S4. Topography of the difference in SDs between self-initiated and externally-triggered conditions. Small circles represent EEG electrodes across which the permutation test was performed. Electrodes that showed significant difference between conditions have been marked *. The time interval (s) is indicated above each subplot.

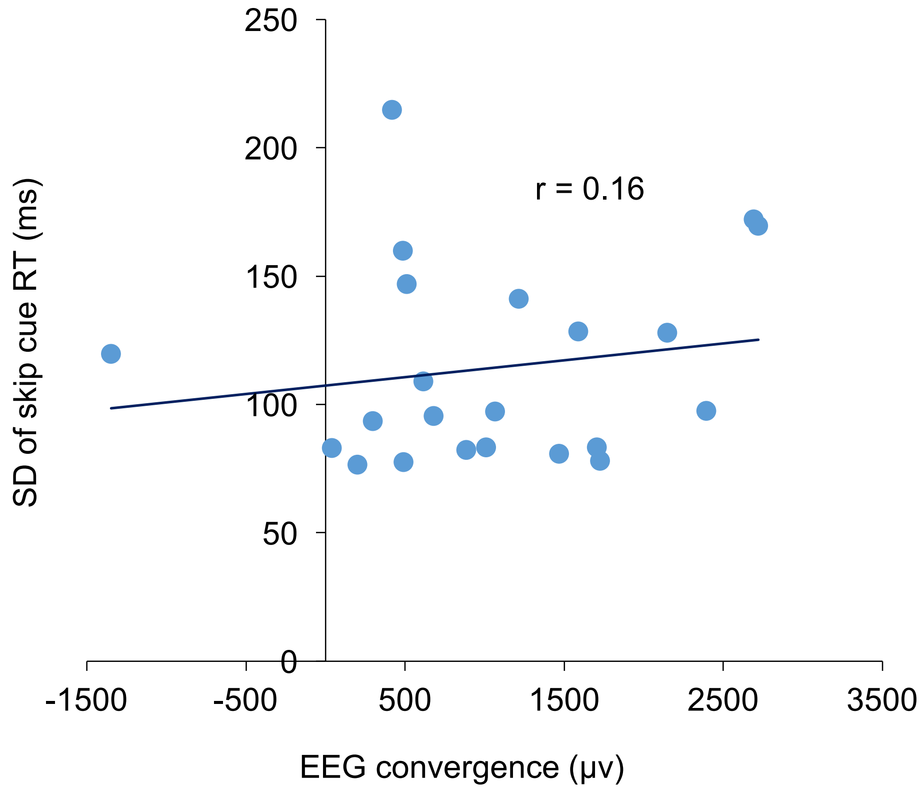

Fig S5. No significant correlation (Pearson's r = 0.16, p = 0.46) across participants between standard deviation of each participant's RT to externally-triggered skip cues (ms), and EEG convergence. EEG convergence was measured by subtracting the area under the SD curve in self-initiated from the externally-triggered condition.

Subject	Mean wait (s) self-initiated	Mean wait (s) externally-triggered	SD (s) self-initiated	SD (s) externally-triggered
1	11.76	12.09	6.82	6.79
2	5.38	5.76	2.27	2.30
3	6.14	6.45	4.58	4.61
4	7.16	7.45	3.25	3.22
5	8.25	8.39	4.57	3.97
6	7.21	7.70	3.22	3.27
7	5.92	6.20	1.66	1.67
8	7.83	8.16	3.20	3.23
9	6.08	6.48	1.26	1.20
10	6.32	6.54	2.96	2.68
11	5.34	5.85	1.00	1.01
12	6.89	7.28	2.72	2.70
13	4.83	5.23	1.83	1.82
14	8.06	8.23	2.66	2.54
15	8.97	9.44	3.60	3.60
16	9.25	9.66	5.85	5.91
17	6.30	6.81	2.69	2.61
18	7.72	8.14	2.53	2.57
19	6.90	7.16	2.94	2.93
20	7.32	7.80	2.40	2.62
21	9.44	9.85	2.93	2.94
22	7.14	7.73	4.74	5.19

Table S1. Mean and standard deviation of waiting time before skipping in self-initiated and externally-triggered conditions.

Drift (I)	Leak (k)	Initial noise (c_1)	Final noise (c_2)	Threshold	Change in noise (Δc)
0.04	-0.18	0.08	0.01	0.10	-0.07
0.11	0.55	0.12	0.13	0.14	0.00
0.11	0.57	0.13	0.11	0.18	-0.02
0.11	0.52	0.12	0.12	0.17	0.00
0.09	0.23	0.19	0.14	0.41	-0.05
0.01	0.38	0.23	0.13	0.42	-0.10
0.10	0.50	0.14	0.13	0.20	-0.01
0.12	0.62	0.10	0.14	0.08	0.04
0.11	0.57	0.11	0.13	0.11	0.01
0.02	0.27	0.25	0.12	0.44	-0.13
0.08	0.14	0.21	0.13	0.38	-0.08
0.11	0.53	0.12	0.12	0.13	0.01
0.09	0.11	0.21	0.12	0.35	-0.09
0.06	0.17	0.21	0.12	0.39	-0.09
0.07	0.28	0.19	0.14	0.36	-0.05
0.11	0.53	0.12	0.11	0.12	-0.01
0.11	0.54	0.12	0.12	0.13	0.01
0.08	0.22	0.18	0.12	0.29	-0.06
0.11	0.60	0.12	0.13	0.09	0.01
0.11	0.53	0.12	0.12	0.13	0.01
0.17	0.59	0.18	0.24	0.74	0.06
0.11	0.55	0.13	0.12	0.15	0.00

Table S2. Optimum parameters for self-initiated skip action. The values were detected by fitting the model against the mean RP amplitude of each participant in self-initiated condition. Δc was measured by subtracting the initial noise level (c_1) from the final noise level (c_2).

Drift (I)	Leak (k)	Initial noise (c_1)	Final noise (c_2)	Threshold	Change in noise (Δc)
0.09	0.42	0.08	0.10	0.10	0.02
0.11	1.01	0.12	0.21	0.14	0.09
0.10	0.35	0.13	0.13	0.18	0.00
0.16	0.70	0.12	0.16	0.17	0.04
0.12	0.48	0.19	0.18	0.41	-0.01
0.10	0.48	0.23	0.17	0.42	-0.05
0.09	0.26	0.14	0.07	0.20	-0.06
0.17	0.93	0.10	0.24	0.08	0.13
0.18	0.44	0.11	0.19	0.11	0.08
0.05	0.44	0.25	0.22	0.44	-0.03
0.10	0.38	0.21	0.17	0.38	-0.04
0.19	0.72	0.12	0.15	0.13	0.03
0.10	0.41	0.21	0.18	0.35	-0.03
0.08	0.54	0.21	0.17	0.39	-0.04
0.12	0.51	0.19	0.23	0.36	0.05
0.16	0.87	0.12	0.15	0.12	0.04
0.18	0.69	0.12	0.16	0.13	0.04
0.10	0.45	0.18	0.15	0.29	-0.03
0.21	0.75	0.12	0.17	0.09	0.05
0.17	0.77	0.12	0.20	0.13	0.09
0.10	0.61	0.18	0.20	0.74	0.01
0.16	0.58	0.13	0.16	0.15	0.03

Table S3. Optimum parameters for externally-triggered skip action. The values were detected by fitting the model against the mean RP amplitude of each participant in externally-triggered condition. Δc was measured by subtracting the initial noise level (c_1) from the final noise level (c_2). c_1 and the threshold were fixed at their optimum values in self-initiated condition (see materials and methods for more details)
